# Regional differences in prediction models of lung function in Germany

**DOI:** 10.1186/1465-9921-11-40

**Published:** 2010-04-22

**Authors:** Eva Schnabel, Chih-Mei Chen, Beate Koch, Stefan Karrasch, Rudolf A Jörres, Torsten Schäfer, Claus Vogelmeier, Ralf Ewert, Christoph Schäper, Henry Völzke, Anne Obst, Stephan B Felix, H-Erich Wichmann, Sven Gläser, Joachim Heinrich

**Affiliations:** 1 Helmholtz Zentrum München, Center for Environmental Health, Institute of Epidemiology, Neuherberg, Germany; 2 Ludwig-Maximilians-University Munich, Dr. von Hauner Children's Hospital, Munich, Germany; 3 University Hospital of the Ernst-Moritz-Arndt University Greifswald, Internal Medicine B, Greifswald, Germany; 4 Helmholtz Zentrum München, Center for Environmental Health, Comprehensive Pneumology Center, Institute of Lung Biology and Diseases, Neuherberg, Germany; 5 Ludwig-Maximilians-University Munich, Institute and Outpatient Clinic for Occupational, Social and Environmental Medicine, Munich, Germany; 6 University Hospital of Giessen and Marburg, Division of Pulmonary Diseases, Marburg, Germany; 7 Ernst-Moritz-Arndt University Greifswald, Insitute for Community Medicine, Greifswald, Germany; 8 Ludwig-Maximilians-University, Institute of Medical Data Management, Biometrics and Epidemiology, Munich, Germany

## Abstract

**Background:**

Little is known about the influencing potential of specific characteristics on lung function in different populations. The aim of this analysis was to determine whether lung function determinants differ between subpopulations within Germany and whether prediction equations developed for one subpopulation are also adequate for another subpopulation.

**Methods:**

Within three studies (KORA C, SHIP-I, ECRHS-I) in different areas of Germany 4059 adults performed lung function tests. The available data consisted of forced expiratory volume in one second, forced vital capacity and peak expiratory flow rate. For each study multivariate regression models were developed to predict lung function and Bland-Altman plots were established to evaluate the agreement between predicted and measured values.

**Results:**

The final regression equations for FEV_1 _and FVC showed adjusted r-square values between 0.65 and 0.75, and for PEF they were between 0.46 and 0.61. In all studies gender, age, height and pack-years were significant determinants, each with a similar effect size. Regarding other predictors there were some, although not statistically significant, differences between the studies. Bland-Altman plots indicated that the regression models for each individual study adequately predict medium (i.e. normal) but not extremely high or low lung function values in the whole study population.

**Conclusions:**

Simple models with gender, age and height explain a substantial part of lung function variance whereas further determinants add less than 5% to the total explained r-squared, at least for FEV1 and FVC. Thus, for different adult subpopulations of Germany one simple model for each lung function measures is still sufficient.

## Background

Spirometric lung function measurements are used for the diagnosis, assessment and management of heart and lung diseases. Furthermore, lung volumes contain prognostic potencies in general populations [1]. In clinical applications, forced expiratory volume in one second (FEV_1_) and forced vital capacity (FVC) are usually expressed as percent-predicted values. To diagnose patients with chronic obstructive lung disease (COPD) or asthma, the Global Initiative for Chronic Obstructive Lung Disease (GOLD) and the Global Initiative for Asthma (GINA) established categories according to FEV_1 _percent-predicted values and the ratios of FEV_1_/FVC [2,3]. In research, FEV_1 _values are also used to normalize the data for known anthropometric determinants [4,5].

Predicted values are commonly derived from measurements performed in a reference population of healthy subjects, stratified according to gender, age and height. To obtain reliable reference values of lung function it is crucial that the reference group is representative and, in particular, free of conditions interfering with the results. Despite these efforts, lung function has a wide variation even in healthy subjects, suggesting that the commonly used determinants do not sufficiently describe the lung function or that the reference populations used differ considerably. In addition to a surprisingly large residual variation, this variation is reflected in differences between currently used reference equations for lung function and discrepancies in international criteria for identifying COPD cases [6-11].

It is well known that in addition to gender, age and height, there are several other factors influencing lung function. The American Thoracic Society (ATS) summarized these sources of variation [12]. Apart from technical factors related to equipment and procedures, biological and environmental factors are among the other possible sources. These include racial and ethnic background; anthropometric and genetic factors; occupational and environmental exposures; nutrition; childhood infections; cardiovascular, metabolic and hormonal disorders; and other factors that have not yet been defined.

In addition, there may be differences in lung function between subpopulations, which should be considered when establishing prediction equations. For example, varying anthropometric characteristics, environmental exposures or social conditions between distinct parts of a country might lead to different reference values.

As a result, the aim of this study was to analyse whether lung function determinants differ between subpopulations within Germany. The comparison of the three studies performed in Germany offers the unique opportunity to analyse whether prediction equations developed for one subpopulation adequately describe lung function for another subpopulation within a country and whether the major determinants of lung function show similar effect size.

## Methods

### Study population

We report the results of three studies which were performed in northern, central and southern areas of Germany. Within the study populations there are no essential differences concerning ethnic background. The majority of participants are Germans (more than 93%).

#### SHIP-I

Within the cross-sectional study SHIP-0 (Study of Health in Pomerania), 4310 individuals, aged 20-79 years, were recruited in the region of Western Pomerania in the Northern part of Germany. Data was collected during 1997 and 2001. Study details are given elsewhere [13]. The 5-year-follow-up SHIP-I, with 3300 participants, was conducted between March 2003 and July 2006. Within the framework of the SHIP-I study lung function tests were performed in 1809 subjects of age 20-85 years. Additional information was evaluated by medical examination and questionnaires.

#### ECRHS-I Erfurt

The cross-sectional study ECRHS-I Erfurt (European Community Respiratory Health Survey) was performed from 1990 to 1992 in Erfurt, Germany; its design has been described in detail [14,15]. In a two-step approach 6291 randomly chosen individuals were asked to reply to a short questionnaire on respiratory symptoms (stage I). Afterwards a population-based random sample of the 4332 stage I responders was invited to attend a clinical examination, perform lung function measurement and answer a detailed questionnaire (stage II). The age range covered subjects between 20 and 65 years. In stage II, 1282 subjects underwent the clinical examination and answered the detailed questionnaire, and 1162 lung function tests were available.

#### KORA C

The community-based study KORA C (Cooperative Research in the Region of Augsburg) is based on the third MONICA survey, which was performed in Augsburg, Germany between 1994 and 1995. The objective and protocol of the MONICA surveys in Augsburg have been published [16]. The third MONICA survey comprised a random sample of 4178 individuals stratified for age and sex, which was drawn from all registered residents of the city of Augsburg aged 25-74 years. KORA C is a subset of the third MONICA survey enriched with subjects with positive testing for specific IgE against common aeroallergens (grass and birch pollen, house-dust mite, cat, and *Cladosporium herbarum*). The KORA C study was performed between September 1997 and December 1998. Details of the KORA C design have been reported [17]. Finally, 1537 subjects participated (60.5%), of whom 50.2% had at least one positive radioallergosorbent test (RAST) result. Besides questionnaire evaluations and medical examinations, lung function tests were performed in 1088 participants who were younger than 60 years.

### Outcome assessment

#### Lung function

All lung function tests were conducted based on the ECRHS protocol [15]. Forced vital capacity (FVC), forced expiratory volume in one second (FEV_1_) and peak expiratory flow rate (PEF) were determined by spirometry in all subjects who did not smoke or use inhalers 1 hour prior to the test. All measured lung function parameters are pre-bronchodilation values. Tests were assumed as valid if at least two technically satisfactory maneuvers were obtained within a maximum of 9 trials. The final values of FVC and FEV_1 _were determined based on the best maneuver as defined by the highest sum of FVC and FEV_1_.

#### Determinants

Anthropometric measurements, computer-assisted standardized interviews and questionnaires were performed in all three studies. The following determinants were taken into account for developing regression models to predict lung function: gender, age, height, weight, obesity, education level, packyears of cigarette smoking for all former and current smokers, environmental tobacco smoke, medication, doctor-diagnosed atopic diseases (asthma), cardiovascular diseases (hypertension, heart attack, stroke) and diabetes. Height and weight were measured and obesity was defined as having a BMI ≥ 30 (body mass index). Education level was defined by the highest graduation: low (less than O-level), medium (O-level) and high (more than O-level). The smoking status (never, current, or former-smoker) and environmental tobacco smoke at home or at work was assessed by self-report. Former-smokers were all people who do not currently smoke but have in the past. The medication was recorded by a computer-aided method using the ATC code. A number of drugs influencing lung function (e.g. sympathomimetic, glucocorticoid, anticholinergic and antiallergenic drugs) were considered for the analysis. Doctor-diagnosed atopic, cardiovascular diseases and diabetes were based on self-report. A positive atopy status was defined as at least one doctor-diagnosed atopic disease.

### Statistical analyses

Descriptive statistical analysis for the study populations and lung function measures was done using SAS, version 9.13, and data was expressed using frequencies, percentages or mean and standard deviation (SD). Chi-square tests and Kruskal-Wallis tests were employed to analyse differences between studies. For each study, multivariate regression models were developed to predict lung function. All predictors were initially entered into the model and the final regression equations were chosen based on the adjusted r-squared value after retaining only the statistically significant predictors. An intracluster correlation of a hierarchical model was used to assess the heterogeneity between the studies. Additionally, Bland-Altman plots were used to evaluate the degree of agreement between the predicted values of the different regression models and the actually measured values.

## Results

For the ECRHS-I Erfurt study lung function test results from 1162 subjects were analysed, for KORA C results from 1088 subjects, and for SHIP-1 results from 1809 subjects (Table 1). There were no major differences between these populations in terms of gender and height. However, the SHIP population had significantly higher values for age (mean ± SD: 52.5 ± 13.7), weight (80.3 ± 15.9) and BMI (27.8 ± 4.7), and it had more than twice as high of an obesity rate (29.1%) in comparison to the two other populations (p < 0.01 for each comparison). The percentage of hypertension and diabetes was highest in the SHIP study, while asthma was more prevalent in the KORA study, which might be due to the study design of KORA C.

**Table 1:** Characteristics of the study populations ECRHS-I, KORA C and SHIP-I

	ECRHS-I (N = 1162)	KORA C (N = 1088)	SHIP-1 (N = 1809)
	n/N (%)	n/N (%)	n/N (%)
Sex (Male)	601/1162 (51.7)	520/1088 (47.8)	885/1809 (48.9)
Age (y)*	41.9 ± 12.3	44.7 ± 9.2	52.5 ± 13.7
Height (cm)	169.8 ± 9.0	170.4 ± 9.3	169.7 ± 9.1
Weight (kg)*	72.9 ± 13.4	74.1 ± 14.2	80.3 ± 15.9
BMI (kg/m^2^)*	25.2 ± 4.0	25.5 ± 4.1	27.8 ± 4.7
Obesity (BMI ≥ 30)*	139/1162 (12.0)	129/1088 (11.9)	526/1809 (29.1)
Education level*			
high	243/1158 (21.0)	251/1069 (23.5)	379/1770 (21.4)
medium	372/1158 (32.1)	295/1069 (27.6)	837/1770 (47.3)
low	543/1158 (46.9)	523/1069 (48.9)	554/1770 (31.3)
Occupation*	818/1162 (70.4)	808/1086 (74.4)	561/1008 (55.7)
Atopic diseases			
Atopy status*	320/1162 (27.5) $	277/1088 (25.5) #	332/1806 (18.4) §
Asthma*	23/1162 (2.0) #	74/1088 (6.8) #	47/1806 (2.6) $
Hay fever*	129/1162 (11.1) $	226/1088 (20.8) #	not available
Cardiovascular diseases			
Hypertension*	265/1066 (24.9) $	231/1086 (21.3) #	668/1807 (37.0) §
Heart attack*	43/1150 (3.7) $	8/1086 (0.7) #	21/1807 (1.2) §
Stroke*	37/1149 (3.2) $	10/1086 (0.9) #	19/1807 (1.1) §
Diabetes*	60/1149 (5.2) $	27/1086 (2.5) #	125/1808 (6.9) §
Menstruation*	287/454 (63.2)	385/568 (67.8)	399/770 (51.8)
Smoking status*			
never	491/1162 (42.3)	425/989 (43.0)	787/1808 (43.5)
current	393/1162 (33.8)	277/989 (28.0)	431/1808 (23.8)
former	278/1162 (23.9)	287/989 (29.0)	590/1808 (32.6)
ETS*	473/1158 (40.9)	287/1088 (26.4)	247/1008 (24.5)
Packyears (y)*	13.2 ± 12.0	13.7 ± 14.5	12.8 ± 12.0
current	15.2 ± 12.3	17.8 ± 14.3	15.0 ± 12.9
former	10.3 ± 11.0	9.8 ± 13.7	11.2 ± 11.1
Medication*	61/1162 (5.2)	66/618 (10.7)	188/1285 (14.6)

Mean ± standard deviation values are given, or proportions and corresponding percentages (in parentheses). Chi-square and Kruskal-Wallis tests were performed to see significant differences; * p < 0.05

Diseases: # ever doctor-diagnosed; §doctor-diagnosed during the last 5 years; $ self-reported, not necessarily doctor-diagnosed; Atopy status: at least one doctor-diagnosed atopic disease

BMI: body mass index; ETS: environmental tobacco smoke; Medication: at least one drug influencing lung function

### Analysis of FEV_1_

Regarding lung function, the SHIP population showed lower FEV_1 _(mean ± SD: 3.5 ± 0.8; p < 0.01 each), FVC (mean ± SD: 3.9 ± 1.0; p < 0.01 each) and PEF values (mean ± SD: 7.3 ± 2.1; p < 0.01 each) compared to the ECRHS and KORA populations (Table 2). However, these differences disappeared when we restricted our analysis to the same age range in all three studies.

**Table 2:** Lung function in the study population

	ECRHS-I (N = 1162)	KORA C (N = 1088)	SHIP-1 (N = 1809)	Total
FEV_1 _(l)	3.6 ± 0.9	3.5 ± 0.8	3.3 ± 0.9	3.4 ± 1.0
FVC (l)	4.4 ± 1.1	4.3 ± 1.1	3.9 ± 1.0	4.1 ± 1.1
PEF (l/s)	8.2 ± 3.1	8.0 ± 2.4	7.3 ± 2.1	7.7 ± 2.5
FEV_1_/FVC	0.81 ± 0.07	0.83 ± 0.08	0.85 ± 0.06	0.83 ± 0.07

Mean values ± standard deviation are given. FEV1: forced expiratory volume in one second; FVC: forced vital capacity; PEF: peak expiratory flow rate;

The final regression equations for FEV_1 _showed adjusted r-squared values between 0.65 and 0.73 (Additional File 1: Table S1). In all three studies, gender, age, height, packyears of cigarette smoking and asthma were significant determinants of FEV_1_, with similar effect sizes in each study. Lower FEV_1 _was associated with female gender, higher age, packyears, and asthma; FEV_1 _increased with height. The negative association with asthma was most pronounced in the ECRHS and SHIP study. Additionally, a low level of education corresponded to lower FEV_1 _in these two studies. Nevertheless, there were differences between the three studies in terms of the other determinants. In KORA environmental tobacco smoke (ETS) and the use of at least one drug influencing lung function was associated with lower FEV_1_, while in the SHIP study obesity was found to have a negative effect on FEV_1_.

### Analysis of FVC

For FVC, adjusted r-squared values ranged between 0.69 and 0.75. Here again gender, age, height and packyears were significant determinants in all three studies (Additional File 1: Table S1). In addition, a low education level was negatively associated with FVC in ECRHS and KORA. This was also the trend in ECRHS and SHIP for subjects with asthma. Obesity and diabetes were linked with low FVC in the SHIP study, while this was true for ETS and the use of medication influencing lung function in the KORA study. However, the effect of diabetes in the SHIP study disappeared when the analysis was restricted to the same age group as in the other studies.

### Analysis of PEF and FEV_1_/FVC

The adjusted r-squared values for PEF ranged between 0.46 and 0.61 (Additional File 1: Table S1). In all three regression models gender, age, height, packyears and education level were significant but the effects of the other determinants varied between the studies. Hypertension was associated with low PEF in the ECRHS study, medication in KORA, and asthma in SHIP. Moreover, PEF slightly increased with weight in the SHIP study.

Regarding the ratio FEV_1_/FVC, adjusted r-squared values ranged between 0.10 and 0.17 (Additional File 1: Table S1). Only gender and height were significant predictors in all studies; age showed an effect only in the ECRHS study. Additionally, weight, asthma, packyears and medication showed very small effects.

### Further analyses

Smoother plots were used to check whether height and weight were linearly associated with lung function. Afterwards a cubic regression model was developed, that confirmed the linear relationship between lung function measures and height and weight. Thus, we did not include higher order terms of height and weight in our analysis.

Furthermore, we compared the model predictivity of our regression models, developed in the current analysis, with standard models that only include gender, age and height as predictors. For all three lung function parameters our models obtained slightly higher adjusted r-squared values compared to the standard models. For example, for FEV_1 _the following adjusted r-squared values could be shown for our models and for the standard models, respectively: 0.72 and 0.69 (ECRHS), 0.65 and 0.63 (KORA), 0.73 and 0.71 (SHIP).

The intracluster correlation of the hierarchical model implied that there is no significant variation between the studies.

**Figure 1:**
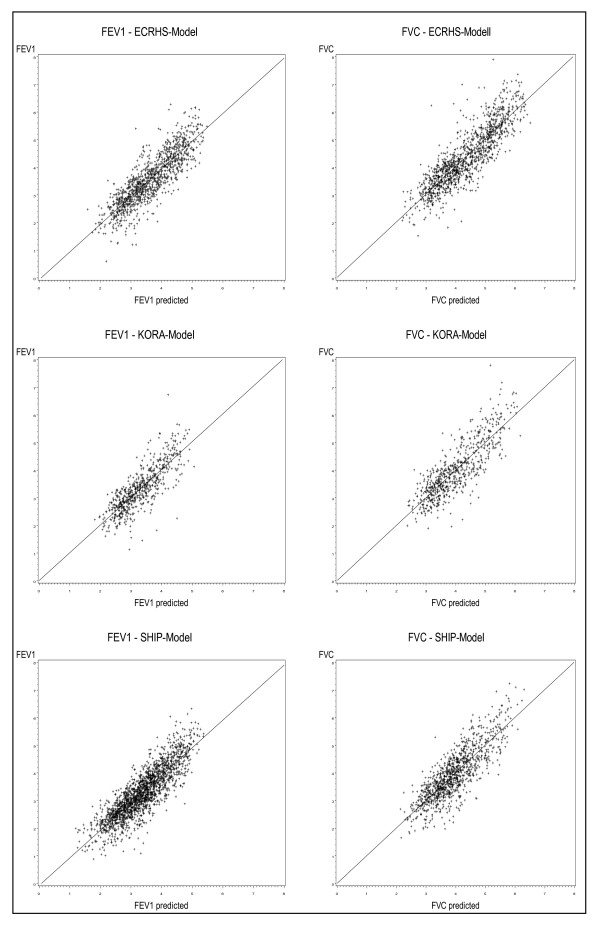
**Measured lung function values versus predicted lung function values for the three regression models**.

The correlation between measured and predicted values is visualised in scatter plots from the regression models of each study (Figure 1). They illustrate a strong correlation between measured and predicted values.

**Figure 2:**
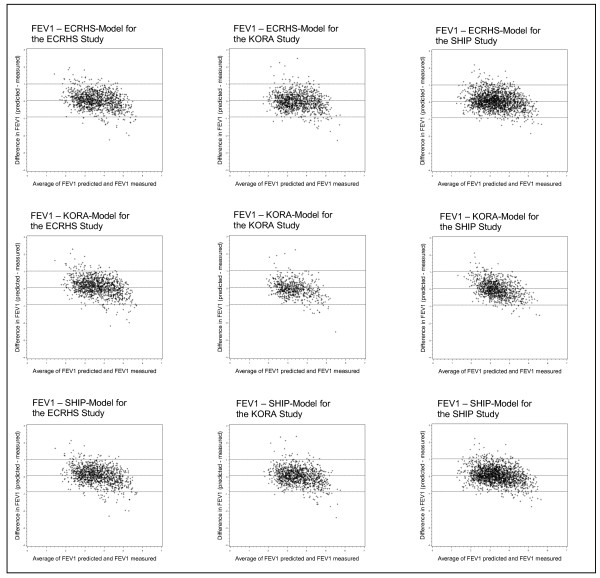
**Bland-Altman Plots for FEV1 stratified for the three studies**.

**Figure 3:**
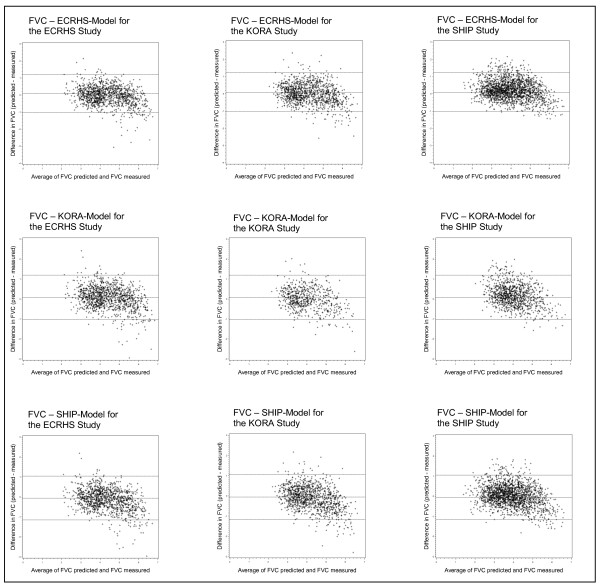
**Bland-Altman Plots for FVC stratified for the three studies**.

Finally, Bland-Altman plots were employed to assess the degree of agreement between predicted and measured values for the three studies (Figure 2 and 3). In all prediction models for FEV_1 _it became apparent that for high FEV_1 _values the predicted FEV_1 _was lower and that for low FEV_1 _values the predicted FEV_1 _was higher than the measured value. For FVC there was a similar trend but the overall model predictivity was less accurate compared to the FEV_1 _prediction model.

## Discussion

The current analysis of three studies performed in South, Central and North Germany confirmed the integrity of the basic determinants of lung function that are usually taken into account in prediction equations. This demonstrates that the data sets used were comparable to data found in the literature. Our analysis revealed that in addition to commonly used predictors such as gender, age and height, several other factors can also be used. These include: the packyears of cigarette smoking, environmental tobacco smoke exposure, the level of education, asthma, body weight, obesity, diabetes, hypertension, and medication. Although there were differences in effect size between the three study populations, these differences were not statistically significant.

Both the European Coal and Steel Community (ECSC) [18] and the American Thoracic Society (ATS) [12] have published comprehensive lists of reference equations for spirometry, and a number of more novel reference equations for different ethical groups and age ranges have been discussed [7-10]. Moreover, the ATS and the European Respiratory Society (ERS) have recently recommended a revision of the reference equations [19]. However, all these reference equations were derived from healthy populations and include only gender, age and height as predictors.

Here, we analyzed a broad panel of potential predictors of lung function in three population-based studies. Using this approach we were not only able to assess whether these factors showed significant associations with lung function, but also whether their contribution to the overall predictive power was significant. On the one hand, this could be relevant to explain differences between prediction equations in different populations, as the additional factors might vary between these populations. On the other hand, a limited size of their additional effect could be the prerequisite for generalizing reference equations from one subpopulation to another or to the general population.

In line with the commonly used reference equations we found that gender, age and height are the major determinants of FEV_1_, FVC and PEF. Additionally, we found that body weight, and the presence of obesity were associated with changes in lung function. Weight itself had only limited effect but regarding obesity the SHIP data demonstrated a considerable reduction of FEV_1 _and FVC. One reason for the strong negative effect might be that in the SHIP study the rate of obesity was twice as high as in the other two studies. Our data are in line previous findings describing an impairment of lung function in subjects that are extremely overweight [12,20,21].

Cigarette smoking is the major risk factor for accelerated lung function decline in adults [22], and it has been demonstrated that the number of cigarettes smoked per day is linearly associated with the rate of decline of lung function [23]. It is also an established result that exposure to ETS has negative effects on respiratory health [24]. ETS exposure has been linked to several diseases, including asthma and COPD, and was demonstrated to be associated with reduced lung function both in children and in adults [12]. In a similar manner our data showed associations between smoking, ETS exposure and lung function impairment. However, the effect of ETS was only detected in the SHIP data, which also showed the highest percentage of ETS exposure.

Socioeconomic status (SES) is another important determinant of lung function and pulmonary diseases [25]. Associations between low SES and lung function, primarily FEV_1 _and FVC, are well known. Smoking contributes to the negative effect of poverty, but other factors are also involved, such as specific environmental and occupational exposures, increased indoor air pollution, low birth weight and increased frequency of respiratory tract infections in childhood [12,25]. Moreover, our analysis revealed a negative correlation between education level and lung function in all three data sets.

Clearly, a number of disorders are also related to impaired lung function. Dyspnoea on exertion, bronchial hyperresponsiveness, asthma and COPD are examples of such conditions [26]. In our study we confirmed asthma to be a strong predictor of impaired lung function, which was statistically significant for almost all lung function measures in the three studies.

Prospective population studies have identified a relationship between low levels of ventilatory function and an increased risk for cardiovascular diseases [26]. FVC and FEV_1 _were inversely related to myocardial infarction [27], and PEF negatively to ischemic heart diseases and stroke [28]. Impaired values of FVC and FEV_1 _were also found in patients with hypertension [29]. Our data are in line with these results, as we confirmed the negative association between hypertension and lung function in the ECRHS study. Cross-sectional studies have also reported negative associations between markers of glucose intolerance and ventilatory function, and FEV_1 _and FVC were inversely correlated to insulin resistance and the prevalence of Type 2 diabetes mellitus [30]. Also, subjects not having the diagnosis of diabetes showed a link between lung function and a raised plasma glucose level [31,32]. Furthermore, we noticed the impairment of lung function in subjects with diabetes in our analysis but this effect became statistically significant only in elderly subjects of the SHIP study.

The multitude of factors potentially influencing lung function is responsible for some of the difficulties in establishing adequate and reliable prediction equations for lung function measures. Taking into account many of these factors, we were able to establish regression models showing a good correlation between predicted and measured lung function values. However, as expected, the major effect was attributable to gender, height and age, which accounted for more than 95% of the variability explained by our regression models. This was true except for in the case of the ratio of FEV1 to FVC, where our regression models could explain only a very small part of the variability. The explanation for this is that the ratio is self-adjusted. This means that the most important determinants for FEV1 and FVC, which are gender, age and height, are cancelled out. This again suggests that simple prediction equations with gender, age and height explain a fair substantial part of lung function variance for FEV1 and FVC. Additionally, the comparison of prediction equations between the three studies pointed out, that each equation was capable of predicting lung function of all three populations with similar accuracy. This suggests that for different subpopulations of Germany a single prediction equation for each of the lung function measures can still be used. However, it should be noted that at the extreme ends of lung function the accuracy of prediction was no longer guaranteed and systematic deviations appeared. One explanation for this lack of agreement might be that beyond the predictors included in our analysis there are additional factors influencing lung function at the extreme ends. There might be specific anthropometric characteristics or exposure to environmental and occupational pollution, including ozone, nitrogen dioxide, sulfur dioxide, dust, chemicals and gases, all of which are known to have adverse effects on lung function [33]. Furthermore, genome-wide studies have revealed that genetic factors have an influence on pulmonary function [34,35], and specific regions on various chromosomes have been identified to be significantly associated with lung function and the occurrence of COPD in smokers [36,37]. However, it has to be considered that these genetic factors only account for a very small proportion (less then 0.15%) of the variance in lung function parameters and that they do not substantially add to clinical variables in predicting the onset of COPD. Another factor related to lung function might be diet. There is a positive association between lung function and the intake of fatty acids or antioxidant vitamins such as vitamin C and E [38,39]. As we did not have information on these factors, further studies taking these into account might be of value.

## Conclusion

Our analysis indicates that in addition to the currently used predictors gender, age and height, other determinants significantly, although to a lower degree, contribute to regression equations for lung function in a general population. The comparison of prediction equations between three studies indicates differences in the patterns of determinants and their effect sizes. However, these differences were not statistically significant, and the effect of the major determinants was similar across the three study areas. This suggests that simple prediction equations with gender, age and height explain a fairly substantial part of lung function variance, at least for FEV1 and FVC, and one simple model for each lung function measure is capable of predicting lung function in all three study areas across Germany.

## Competing interests

The authors declare that they have no competing interests.

## Authors' contributions

ES was responsible for the data analysis, interpretation of data and manuscript preparation. C-MC and JH assisted in the data analysis, interpretation and critical reversion of the results. TS was responsible for lung function measurements. BK, SK, RAJ, SG and JH assisted in the critical revision of the manuscript. CV, RE, CS, HV, AO, SBF, H-EW, SG and JH were responsible for the data. All authors read and approved the final manuscript. The ECRHS, the KORA and the SHIP study group were responsible for the design and conduct of the three studies.

## Supplementary Material

Additional file 1 **Table S1: Regression models for predicting FEV1, FVC, PEF and FEV1/FVC in the ECRHS-I, KORA C and SHIP-I study**. Only the statistically significant terms were retained in the regression models and are shown. SD: standard deviation; R^2^: adjusted R-squared; Gender (Female); Education level: 1 = medium, 2 = high; ETS: environmental tobacco smoke. Click here for file
