# Supplementary material for: Regional differences in prediction models of lung function in Germany
Source: Respir Res. 2010 Apr 22;11(1):40. doi: 10.1186/1465-9921-11-40 (PMC2873930; doi:10.1186/1465-9921-11-40)
Supplement: Additional file 1 — Table S1: Regression models for predicting FEV1, FVC, PEF and FEV1/FVC in the ECRHS-I, KORA C and SHIP-I study. Only the statistically significant terms were retained in the regression models and are shown. SD: standard deviation; R2: adjusted R-squared; Gender (Female); Education level: 1 = medium, 2 = high; ETS: environmental tobacco smoke. [file 1465-9921-11-40-S1.DOC]

**Table S1: Regression models for predicting FEV_1_, FVC, PEF and FEV_1_/FVC in the ECRHS-I, KORA C and SHIP-I study**

| **ECRHS-I** | | | | |  | **KORA C** | | | | |  | **SHIP-I** | | | | |
| --- | --- | --- | --- | --- | --- | --- | --- | --- | --- | --- | --- | --- | --- | --- | --- | --- |
| **Variable** | **Estimate** | **SD** | **P-Value** | **R²** |  | **Variable** | **Estimate** | **SD RSD SEE** | **P-Value** | **R²** |  | **Variable** | **Estimate** | **SD** | **P-Value** | **R²** |
| **FEV_1_** |  |  |  |  |  | **FEV_1_** |  |  |  |  |  | **FEV_1_** |  |  |  |  |
| Constant | -0.74 | 0.47 | 0.12 | 0.722 |  | Constant | -0.72 | 0.64 | 0.26 | 0.654 |  | Constant | -1.87 | 0.37 | <0.01 | 0.732 |
| Gender | -0.69 | 0.04 | <0.01 |  |  | Gender | -0.59 | 0.06 | <0.01 |  |  | Gender | -0.50 | 0.03 | <0.01 |  |
| Age (y) | -0.03 | 0.001 | <0.01 |  |  | Age (y) | -0.03 | 0.0021 | <0.01 |  |  | Age (y) | -0.03 | 0.001 | <0.01 |  |
| Heigth (cm) | 0.04 | 0.003 | <0.01 |  |  | Heigth (cm) | 0.04 | 0.0031 | <0.01 |  |  | Heigth (cm) | 0.04 | 0.002 | <0.01 |  |
| Weight (kg) | 0.003 | 0.001 | 0.02 |  |  | Asthma | -0.19 | 0.08 | 0.01 |  |  | Obesity | -0.09 | 0.02 | <0.01 |  |
| Education 1 | 0.06 | 0.04 | 0.10 |  |  | Packyears (y) | -0.01 | 0.0015 | <0.01 |  |  | Education 1 | 0.07 | 0.03 | 0.02 |  |
| Education 2 | 0.19 | 0.04 | <0.01 |  |  | ETS | -0.08 | 0.04 | 0.06 |  |  | Education 2 | 0.07 | 0.03 | 0.04 |  |
| Asthma | -0.48 | 0.10 | <0.01 |  |  | Medication | -0.21 | 0.07 | <0.01 |  |  | Asthma | -0.40 | 0.07 | <0.01 |  |
| Packyears (y) | -0.01 | 0.001 | <0.01 |  |  |  |  |  |  |  |  | Packyears (y) | -0.01 | 0.001 | <0.01 |  |
|  |  |  |  |  |  |  |  |  |  |  |  |  |  |  |  |  |
|  |  |  |  |  |  |  |  |  |  |  |  |  |  |  |  |  |
| **FVC** |  |  |  |  |  | **FVC** |  |  |  |  |  | **FVC** |  |  |  |  |
| Constant | -2.72 | 0.51 | <0.01 | 0.742 |  | Constant | -2.66 | 0.75 | <0.01 | 0.691 |  | Constant | -2.74 | 0.41 | <0.01 | 0.747 |
| Gender | -0.77 | 0.05 | <0.01 |  |  | Gender | -0.72 | 0.07 | <0.01 |  |  | Gender | -0.60 | 0.04 | <0.01 |  |
| Age (y) | -0.02 | 0.002 | <0.01 |  |  | Age (y) | -0.03 | 0.0025 | <0.01 |  |  | Age (y) | -0.03 | 0.001 | <0.01 |  |
| Heigth (cm) | 0.05 | 0.003 | <0.01 |  |  | Heigth (cm) | 0.06 | 0.0037 | <0.01 |  |  | Heigth (cm) | 0.05 | 0.002 | <0.01 |  |
| Education 1 | 0.04 | 0.04 | 0.37 |  |  | Education 1 | 0.04 | 0.05 | 0.47 |  |  | Obesity | -0.14 | 0.03 | <0.01 |  |
| Education 2 | 0.25 | 0.04 | <0.01 |  |  | Education 2 | 0.12 | 0.06 | 0.05 |  |  | Asthma | -0.20 | 0.08 | 0.01 |  |
| Asthma | -0.36 | 0.11 | <0.01 |  |  | Packyears (y) | -0.01 | 0.0018 | <0.01 |  |  | Diabetes | -0.13 | 0.05 | 0.01 |  |
| Packyears (y) | -0.01 | 0.002 | <0.01 |  |  | ETS | -0.11 | 0.05 | 0.04 |  |  | Packyears (y) | -0.01 | 0.001 | <0.01 |  |
|  |  |  |  |  |  | Medication | -0.21 | 0.07 | <0.01 |  |  |  |  |  |  |  |
|  |  |  |  |  |  |  |  |  |  |  |  |  |  |  |  |  |
|  |  |  |  |  |  |  |  |  |  |  |  |  |  |  |  |  |
| **PEF** |  |  |  |  |  | **PEF** |  |  |  |  |  | **PEF** |  |  |  |  |
| Constant | 3.81 | 1.94 | 0.05 | 0.573 |  | Constant | -0.49 | 2.30 | 0.83 | 0.458 |  | Constant | 0.04 | 1.06 | 0.97 | 0.606 |
| Gender | -3.57 | 0.18 | <0.01 |  |  | Gender | -2.02 | 0.22 | <0.01 |  |  | Gender | -1.88 | 0.09 | <0.01 |  |
| Age (y) | -0.03 | 0.01 | <0.01 |  |  | Age (y) | -0.03 | 0.01 | <0.01 |  |  | Age (y) | -0.04 | 0.003 | <0.01 |  |
| Heigth (cm) | 0.06 | 0.01 | <0.01 |  |  | Heigth (cm) (cm)th | 0.08 | 0.01 | <0.01 |  |  | Heigth (cm) | 0.07 | 0.01 | <0.01 |  |
| Education 1 | 0.24 | 0.16 | 0.13 |  |  | Education 1 | 0.24 | 0.17 | 0.16 |  |  | Weight (kg) | 0.01 | 0.002 | 0.04 |  |
| Education 2 | 0.57 | 0.17 | <0.01 |  |  | Education 2 | 0.37 | 0.18 | 0.04 |  |  | Education 1 | 0.30 | 0.08 | <0.01 |  |
| Hypertension | -0.34 | 0.15 | 0.02 |  |  | Packyears (y) | -0.02 | 0.01 | <0.01 |  |  | Education 2 | 0.43 | 0.09 | <0.01 |  |
| Packyears (y) | -0.03 | 0.01 | <0.01 |  |  | Medication | -0.48 | 0.22 | 0.03 |  |  | Asthma | -0.53 | 0.20 | 0.01 |  |
|  |  |  |  |  |  |  |  |  |  |  |  | Packyears (y) | -0.02 | 0.003 | <0.01 |  |
|  |  |  |  |  |  |  |  |  |  |  |  |  |  |  |  |  |
|  |  |  |  |  |  |  |  |  |  |  |  |  |  |  |  |  |
| **FEV_1_/FVC** |  |  |  |  |  | **FEV_1_/FVC** |  |  |  |  |  | **FEV_1_/FVC** |  |  |  |  |
| Constant | 1.18 | 0.06 | <0.01 | 0.169 |  | Constant | 1.24 | 0.06 | <0.01 | 0.098 |  | Constant | 1.10 | 0.04 | <0.01 | 0.169 |
| Gender | -0.02 | 0.01 | 0.01 |  |  | Gender | -0.002 | 0.0003 | <0.01 |  |  | Gender | -0.001 | 0.0001 | <0.01 |  |
| Age (y) | -0.002 | 0.0002 | <0.01 |  |  | Heigth (cm) | -0.002 | 0.0003 | <0.01 |  |  | Heigth (cm) | -0.001 | 0.0002 | <0.01 |  |
| Heigth (cm) | -0.002 | 0.0004 | <0.01 |  |  | Medication | -0.03 | 0.01 | <0.01 |  |  | Weight (kg) | 0.001 | 0.0001 | <0.01 |  |
| Weight (kg) | 0.001 | 0.0002 | <0.01 |  |  |  |  |  |  |  |  | Asthma | -0.06 | 0.01 | <0.01 |  |
| Asthma | -0.05 | 0.01 | <0.01 |  |  |  |  |  |  |  |  | Packyears (y) | -0.001 | 0.0002 | <0.01 |  |
| Packyears (y) | -0.001 | 0.0002 | <0.01 |  |  |  |  |  |  |  |  | Medication | -0.02 | 0.01 | <0.01 |  |
| Medication | -0.02 | 0.01 | 0.04 |  |  |  |  |  |  |  |  |  |  |  |  |  |

Only the statistically significant terms were retained in the regression models and are shown. SD: standard deviation; R²: adjusted R-squared; Gender (Female); Education level: 1=medium, 2=high; ETS: environmental tobacco smoke
